# Therapeutic Targeting of Neutrophil Granulocytes in Inflammatory Liver Disease

**DOI:** 10.3389/fimmu.2019.02257

**Published:** 2019-09-20

**Authors:** Matthias Bartneck, Jing Wang

**Affiliations:** ^1^Department of Medicine III, Medical Faculty, Rheinisch-Westfälische Technische Hochschule Aachen, Aachen, Germany; ^2^Shanghai Institute of Immunology, Shanghai Jiao Tong University School of Medicine, Shanghai, China

**Keywords:** neutrophil granulocytes, macrophages, small non-coding RNA, micro-RNA, small interfering RNA, inflammatory liver disease, two-photon microscopy, intravital imaging

## Abstract

Neutrophil granulocytes are the most numerous type of leukocyte in humans bearing an enormous, yet largely unexplored therapeutic potential. Scientists have very recently increased their efforts to study and understand these cells which contribute to various types of inflammatory diseases and cancer. The mechanisms that regulate neutrophil recruitment to inflamed tissues and neutrophil cytotoxic activities against host tissues and pathogens require more attention. The reactive oxygen species (ROS) are a popular source of cellular stress and organ injury, and are critically expressed by neutrophils. By combating pathogens using molecular combat factors such as neutrophil extracellular traps (NETs), these are immobilized and killed i.e., by ROS. NETs and ROS are essential for the immune defense, but upon excessive activation, may also harm healthy tissue. Thus, exploring new routes for modulating their migration and activation is highly desired for creating novel anti-inflammatory treatment options. Leukocyte transmigration represents a key process for inflammatory cell infiltration to injury sites. In this review, we briefly summarize the differentiation and roles of neutrophils, with a spotlight on intravital imaging. We further discuss the potential of nanomedicines, i.e., selectin mimetics to target cell migration and influence liver disease outcome in animal models. Novel perspectives further arise from formulations of the wide array of options of small non-coding RNA such as small interfering RNA (siRNA) and micro-RNA (miR) which exhibit enzymatic functions: while siRNA binds and degrades a single mRNA based on full complementarity of binding, miR can up and down-regulate multiple targets in gene transcription and translation, mediated by partial complementarity of binding. Notably, miR is known to regulate at least 60% of the protein-coding genes and thus includes a potent strategy for a large number of targets in neutrophils. Nanomedicines can combine properties of different drugs in a single formulation, i.e., combining surface functionalization with ligands and drug delivery. Inevitably, nanomedicines accumulate in other phagocytes, a fact that should be controlled for every novel formulation to restrain activation of macrophages or modifications of the immunological synapse. Controlled drug release enabled by nanotechnological delivery systems may advance the options of modulating neutrophil activation and migration.

## Introduction

### Origin, Roles, and Differentiation of Neutrophil Granulocytes

Neutrophil granulocytes are the most numerous innate immune cell type of and represent 50–70% of the circulating leukocytes. Under homeostatic conditions, neutrophils are generated in the bone marrow at a rate of 10^9^ cells per kilogram of body weight per day (1). A plethora of factors controls the differentiation of hematopoietic cells into myeloblasts, metamyelocytes, band cells, and finally, granulocytes (2). Neutrophils play an important role in the defense of the body, as well as in resolution and healing of inflammation (3). Neutrophils phagocytose and digest pathogens, and further kill microbes based on a repertoire of antimicrobial molecules, some of which they store in their repository granules and which they can release toward microbes upon activation (4). The granules, which are a major trait of these cells, contain among others different types of proteins which can be classified as azurophilic, specific, and gelatinase granules. Azurophilic granules mainly include myeloperoxidase (MPO), the enzyme which mediates the oxidative burst (5, 6). Specific granules contain lactoferrin and get generated after the generation of azurophilic granules. The gelatinase granules are loaded with antimicrobial agents and function as a deposit for diverse metalloproteases, such as gelatinase, and leukolysin. The granules thus cover a collection of toxic substances which are required for the diverse activities of neutrophils during inflammation and which impact innate and adaptive immunity (7).

Neutrophils are normally terminally differentiated when entering the bloodstream, and they exhibit a short life span of only 6–8 h in circulation (8). Neutrophils are distinguished as either being circulating or belonging to the marginated pool. The cells of the marginated pool are those cells which reside in liver, spleen, and bone marrow and which can be re-mobilized into the circulation by specific signals, for instance, adrenaline (9, 10). The life of neutrophils is strongly controlled by inflammatory signals which “keep them alive.” In case these signals are lacking, neutrophils die by spontaneous apoptosis. The clearance of dead neutrophils takes place in the liver, spleen, and bone marrow (10). The life span is thus tightly regulated during inflammatory responses, and the turnover rate of these cells can be delayed or accelerated (11, 12). The diversity of neutrophil subtypes can in parts be explained by their level of maturation: “fresh” neutrophils upregulate surface markers such as the CXC chemokine receptor 2 (CXCR2), Gr1 (Ly6C/G), and L-selectin (CD62L) (13). Earlier studies have outlined the importance of CXCR2 upon thermal injury and emphasized the role of the CXCL2/CXCL1-CXCR2 axis in murine neutrophil intravascular chemotaxis toward injury sites (14).

**Figure 1:**
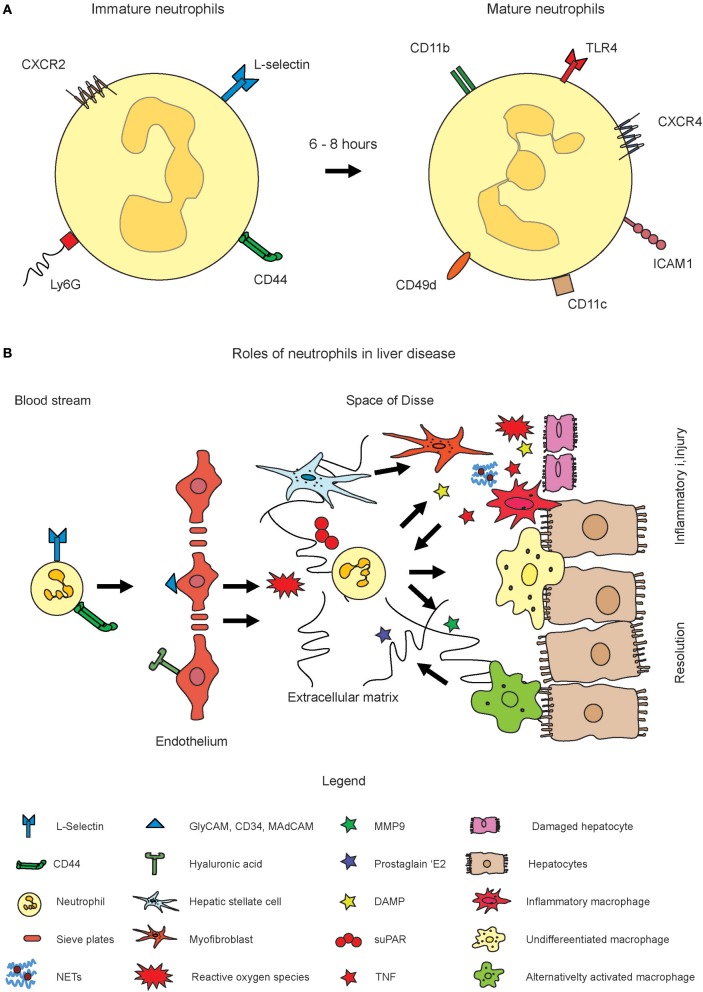
Role of neutrophil activation in inflammatory liver disease. Neutrophils originate from hematopoietic stem cells in the bone marrow. **(A)** Fresh neutrophils exhibit a more rod-like morphology of the nucleus and express CXCR2, L-Selectin, and Ly6G on the surface (left cell). Mature neutrophils express markers such as CXCR4, ICAM1, CD11c, and others and exhibit a segmented nucleus. Please note that the expression of surface markers is highly dynamic. The markers displayed in the figure rather indicate shifts toward higher or lower expression of the markers. **(B)** Upon inflammation, their numbers in circulation largely increase and they infiltrate inflamed tissue sites through guidance by endothelial signal molecules such as selectins or hyaluronic acid. Upon activation, reactive oxygen species (ROS) and neutrophil extracellular traps (NET) get formed which are intended to combat pathogens. The ROS and NETs may also harm healthy tissue or amplify inflammation. MMP9 and also ROS were also shown to induce resolution of inflammation and to trigger alternative activation of macrophages.

The retention and release of neutrophils from the bone marrow is regulated by CXCR4 and CXCR2. Aged neutrophils, after 6–8 h, among others express CXCR4, intercellular adhesion molecule 1 (ICAM1), and CD49d (13). The integrin α4β1 (VLA4) and the CXC chemokine receptor 4 (CXCR4) are downregulated, while CXCR2 and Toll-like receptor 4 (TLR4) are upregulated on immature neutrophils (15). The CXCR4 is of critical importance for neutrophil maintenance in the bone marrow. It has been shown that deficiency in CXCR4 causes changes in the populations of mature neutrophils in circulation. The stromal cell-derived factor 1 (SDF-1, CXCL12) is strongly expressed by bone marrow endothelial cells as well as osteoblasts and binds to CXCR4, and the CXCR4/SDF-1 signal axis is required to sustain the neutrophils in the bone marrow. Appropriate signals can rapidly recruit neutrophils if required (16, 17). Upon blocking CXCR4, neutrophilia is induced as a consequence of bone marrow egress. In addition, neutrophil pools from other organs such as the lung can also be mobilized into circulation (18). The mobilization of neutrophils can also occur through the granulocyte colony stimulating factor (G-SCF, CSF3), which is known to switch the balance between CXCR2 and CXCR4 toward CXCR2. It is assumed that this is based on downregulating CXCL12 expression and by the induction of CXCL1 and CXCL2 expression in the endothelial cells of the bone marrow (19). The toll-like receptor 4 (TLR4) triggers the release of neutrophil extracellular traps (NET) (see below, more details in section Recruitment and Functions of Neutrophil Granulocytes in Acute Liver Disease) (20). Neutrophils were shown to express CD11c, which is normally a marker for dendritic cells, in systemic inflammatory response syndrome (SIRS) patients (21). In addition to the surface receptors, the cellular nucleus undergoes characteristic during neutrophil maturation. It changes from a round into a banded shape with lobulated morphology. These changes in the core are also used to assess immature and mature neutrophils in blood sample tests for inflammation where an increased number of round or rod-like core shape reflects a “left switch” with increased numbers of immature neutrophils. Importantly, the expression of the surface markers by neutrophils are dynamic and should be considered as either higher or lower, rather than positive or negative (Figure 1A).

The concept of neutrophil heterogeneity in both basal and inflammatory conditions has become an emerging focus. Neutrophil subsets were initially defined in 2004 outlining the existence of three populations on the basis of their expression of TLR, CD49d, or CD11b (22). The cell surface glycoprotein CD177 is another marker for neutrophils which is expressed on 40–60% of peripheral neutrophils under normal conditions and it is upregulated during bacterial infection or inflammation. The CD177^+^ neutrophils were demonstrated to generate low levels of inflammatory cytokines, but exhibit increased bactericidal activity and to produce huge amounts of reactive oxygen species (ROS), myeloperoxidase and NETs in a colitis model (23). The so-called low-density granulocytes are proficient in generating NETs and were described as pro-inflammatory neutrophil subsets (24, 25), which can easily be observed in flow cytometry without the need of antibody staining. Olfactomedin 4 (OLFM4), a matrix glycoprotein stored in specific granules is another marker which expression can be induced by NETs. It is expressed by ~10–30% of the circulating neutrophils. Data indicating the subpopulations are currently still vague, but the existence of two subpopulations is suggested (26, 27).

However, as mentioned above, many neutrophil subsets that are described in the literature might only represent neutrophils in different activation, polarization, or maturation states instead of truly subsets that have distinguished transcriptional programs and functions. Nevertheless, an attractive therapeutic potential has been proposed by specific targeting of neutrophil subsets in pathological conditions. However, modulation of neutrophils can exhibit side effects such as increased risk of infection. Moreover, the exact roles of neutrophil subsets in the liver are not clear. Liver is known as a marginated pool of neutrophils, in which these cells adhere to the endothelium of capillaries and postcapillary venules, resulting in a prolonged transition time when passing through this organ. Thus, it is an intriguing question whether neutrophils change their phenotype in liver in response to local vs. systemic cues especially under pathologic conditions.

### Recruitment and Functions of Neutrophil Granulocytes in Acute Liver Disease

Inflammatory reactions at tissue sites are frequently initiated by macrophages which recognize pathogens by means of their expression of pattern recognition receptors, in particular, TLR. TLR4 probably is the most intensively studied receptor of the family and is involved in the recognition of bacterial products such as lipopolysaccharides (LPS) (28). One major consequence of macrophage activation is their release of the tumor necrosis factor (TNF) which plays a key role in many types of inflammatory diseases. Other cytokines secreted by macrophages and other cell types include neutrophil attractants which bind to receptors on neutrophils. For instance, the stromal derived factor 1 (SDF-1, CXCL12) for CXCR4, and CXCL1 (also KC or Groβ), which binds to CXCR2, thus have an impact on neutrophil activation and migration (13).

In general, neutrophil recruitment into different organs has major similarities: it is a sequential event which includes steps of tethering, rolling, adhesion, crawling, and transmigration (29–31). In this process, the stimulation of endothelial cells by inflammatory factors such as the TNF triggers them to express P and E selectins, adhesion molecules which control leukocyte transmigration (diapedesis). Neutrophils constitutively express the corresponding glycosylated ligands such as the P-selectin glycoprotein ligand 1 (PSGL-1). The anchoring of the ligands to the selectins evokes the tethering of neutrophils to the endothelium layer. Tethering is followed by rolling alongside the direction of the blood flow. After leukocyte rolling, integrins get activated in response to tissue-derived chemokines which get sequestered in the endothelial glycocalyx (32). The formation of tethers with the endothelium further fixes their adhesion to the blood flow (33). The binding of the ligands to PSGL-1 activates a variety of kinases, i.e., of the p38 mitogen-activated protein kinase, which is a major prerequisite for integrin activation and adhesion (34, 35). During the rolling process, β2-integrins of neutrophils undergo a conformational change, making them more adhesive to their ligand intracellular adhesion molecule 1 (ICAM-1), which is expressed by activated endothelium (36). Rolling enables a firm adhesion to the endothelium and is followed by flattening of the cells. Subsequently, leukocytes crawl on top of the endothelial apical surface to locate a site for extravasation (37). The adhesion to the endothelium is followed by an either transcellular migration through or between interendothelial junctions (38). During transmigration, molecules such as junctional adhesion molecules A, B and C (JAM-A, JAM-B, and JAM-C), which are of major importance due to their interactions with the integrin Macrophage-1 antigen (integrin αMβ2/macrophage integrin/Mac-1) (39). Subsequently, neutrophils have to migrate through pericytes which reside on top of the endothelial cell layer.

In most organs, a basement membrane is the next barrier to cross and it is a mesh composed of laminins and collagens. The venular pericytes support the crawling of neutrophils during their egress from the vessel lumen (40). However, unlike other types of fenestrated endothelium, i.e., that of the kidney, hepatic fenestrations exhibit no diaphragm and also no basal lamina. The endothelial cells are organized into sieve plates, what leads to a comparatively high level of permeability of LSEC (41). but liver fibrosis is known to lead to an accumulation with extracellular matrix (ECM) in the space of Disse (42). The fenestrations of LSEC were demonstrated to exhibit a filter function that determines which type of macromolecules can reach the parenchymal cells (43). In many other organs, neutrophils are exposed to a high level of shear stress which they can adapt by changing toward a flat morphology. However, in liver, where the infiltration occurs within the narrow sinusoidal channels (44), which in some areas only exhibit the size of a flowing leukocyte, the shear stress is rather low (41). This fact reduces the necessity of the rolling step, and also relates to the low expression of selectins (44). The transmigration into liver has more organ-specific properties: for instance, recruitment into the hepatic sinusoids does not require the rolling step; instead, neutrophils adhere directly to the hepatic endothelium (45). In addition, selectin dependency of the recruitment of neutrophils into the liver is controversial. It was reported as early as 1997 that, under inflammatory conditions, the sinusoids are the major entry site for neutrophils (80%), whereas a smaller portion of cells adheres to the post-sinusoidal venules (44). Particularly, adhesion to the sinusoids is almost unaffected by selectins as demonstrated by using selectin deficient mice and selectin inhibitors (44). Furthermore, also other adhesion molecules were reported to play minor roles for the liver entry, including α2 integrin and ICAM-1 (44).

The group of Paul Kubes demonstrated that neutrophils express CD44 on their surface to bind hyaluronan expressed by the LSEC to enter inflamed liver microvasculature (46). McDonald and colleagues have demonstrated that the activation of TLR4 on LSEC triggered the deposition of serum-associated hyaluronan-associated protein. Neutrophils also express CD44, a cellular membrane glycoprotein which is involved in cell–cell interactions, cell adhesion, and migration. It is a receptor for hyaluronic acid (HA) expressed by liver sinusoidal cells (LSEC), and further interacts with osteopontin, collagens, and matrix metalloproteinases (MMP), thereby facilitates interactions with the extracellular matrix (ECM). The CD44-HA interaction mediates neutrophil adhesion to liver sinusoidal cells (LSEC) and thus represents a mechanism for transmigration (47). In sterile rodent injury models such as thermal injury, neutrophil recruitment is less dependent on HA-CD44, but the cells use αMβ2 (Mac-1) in order to bind to ICAM1 (48). Importantly, this binding mechanism is not important in sepsis models in which IL10 is found in serum at high concentration, where IL10 strongly reduces the expression of αMβ2 (49). During septic injury, infiltrating neutrophils readily arrest upon entry, whereas in sterile injury, the cells migrate toward the focus of damage and may adapt the so-called swarming behavior which is amplified by leukotriene B4 (LTB4) as described by Tim Lämmermann and coworkers (50). During sterile injury, neutrophil recruitment can be enhanced by adenosine triphosphate (ATP) which is released from necrotic hepatocytes (48). Similarly, dying and dead hepatocytes release N-formyl peptides which are detected by the formylated receptor 1 on neutrophils, and which thereby guide the cells to the specific location of injury (48). These complementing mechanisms allow neutrophils to adapt to different stages and modes of inflammation. For instance, they are thus able to prioritize their response toward necrotic areas, whereas the chemokine-directed migration has a lower priority (51). A mode of rapid migration of neutrophils was reported to be triggered by myosin heavy chain 9 (Myh9) which is located at lamellopodes and in the uropod. In laser-induced skin injury and in acute peritonitis, reduced Myh9 expression in the hematopoietic system resulted in significantly diminished neutrophil extravasation (52). The accumulation of neutrophils in the microvasculature of the liver is a key pathological feature of the systemic inflammatory response to sepsis or endotoxemia. In this disease, neutrophils cause tissue damage and vascular dysfunction. In addition to the TLR4 activation of the endothelium, Kupffer cells impact neutrophil recruitment based on a specific mechanism which is independent of CD44, HA, and (serum-derived hyaluronan-associated protein) SHAP (53). It was reported that a signaling network of TLR2, S100A9, and CXCL2 is required to recruit neutrophils in acute (and chronic) liver injury based on carbon tetrachloride (CCl_4_)-mediated toxic liver injury (54).

In addition to their comparatively well-studied interactions with the endothelium, neutrophils also perform important interactions with platelets. Neutrophils and platelets similarly are equipped with readily available functional molecules and combine their activities to shapen the early inflammatory response, as reviewed in great detail before (55). Following their activation, platelets release damage-associated molecular patterns (DAMP) such as High-Mobility-Group-Protein B1 (HMGB1). Notably, HMGB1 can form heterocomplexes with CXCL12 and is thus capable to engage CXCR4 in order to initiate neutrophil recruitment to sites of inflammation (56). Platelets function as pathfinders for neutrophils in the microvasculature. The initiation of inflammation is followed by the adherence of circulating platelets to distinct sites in venular microvessels, thereby capturing neutrophils. Subsequently, inflammatory monocytes get recruited via CD40-CD40L interactions (57).

Thus, in summary, the mechanisms for neutrophil recruitment are strictly context-dependent and highly dynamic, thus they depend on the specific type of disease (model) and of associated cell types. For example, Mac-1 is required for neutrophil adhesion and crawling during local inflammatory stimuli in sinusoids as mediated by thermal injury, whereas in systemic inflammatory disease conditions, the exposure to IL10 evokes a CD44-dependent, integrin-independent adhesion (49). The infiltration of the liver or other organs with neutrophils leads to a potential accumulation also with the different compounds secreted by neutrophils, and these may affect disease outcome, i.e., by harming healthy tissue. These cells are known to produce reactive oxygen species (ROS), and degranulate many other compounds such as the sticky web-like structures composed of decondensed chromatin filaments and proteins termed neutrophil extracellular traps (NETs) (58). Neutrophil elastase (NE) is involved in chromatin decondensation occurring in NETs. ROS and NETs function mainly in combating pathogens, yet an excessive production may lead to an amplification of the inflammatory response which, if unresolved, can lead to unspecific tissue damage (59). Pro-inflammatory properties and contributions to host cell injury have been reported for NETs and were proposed to contribute to many types of disease. NETs were demonstrated to contribute to inflammation and tissue damage in mice with severe acute pancreatitis (60). In diabetes, neutrophils are primed to undergo NETosis, which in turn impairs wound healing. Consequently, wound healing is accelerated in peptidylarginine deiminase 4 (Pad4) deficient mice (which have a deficiency in NET formation) compared to WT mice. NETs enriched in oxidized mitochondrial DNA are further interferogenic and contribute to lupus-like disease. A recent report revealed that the nuclear protein histone H4 that is released with NETs binds to and lyses smooth muscle cells (SMC) thus contributing to arterial tissue damage (61).

Neutrophils also secrete proteins which can systemically amplify inflammatory reactions. For instance, the soluble urokinase plasminogen activator receptor (suPAR) received increasing attention, since it is closely associated with the severity of systemic inflammation and has been linked to immune activation in critically ill patients. Similar to its prognostic properties in patients with sepsis or cirrhosis, intrahepatic uPAR activation and serum suPAR concentrations serve as an interesting biomarker for acute liver failure patients (62). The suPAR was originally identified to be generated by bone-marrow derived immature myeloid cells and to contribute to proteinuric kidney disease (63). Recently, our own studies on blood cells and serum from critically ill patients have revealed low uPAR expression on neutrophil surface and high serum suPAR levels. This inverse correlation supports the notion that neutrophils are a main source of shed suPAR proteins in systemic inflammation. Furthermore, high suPAR levels and low neutrophilic uPAR expression predict mortality in ICU patients (64). Open questions remain from studies in which the role of monocytic and resident macrophages has been studied in acetaminophen (Paracetamol, abbreviated as APAP)-induced liver injury. In this type of acute liver injury, neutrophils appear at high numbers of about 20–30% of leukocytes, prior to the monocytic cells (14).

However, neutrophil effectors do not necessarily damage tissue, neutrophils can also contribute to the resolution of inflammation. It has for instance been reported that myeloperoxidase (MPO) can also protect the host from lipopolysaccharide (LPS)-induced tissue injury (65). In thermal injury, neutrophils penetrate the site of injury site and perform the important task of dismantling injured vessels and creating channels for vascular renewal. Subsequently, neutrophils reenter the vasculature and perform a trip that covers a sojourn in the lungs which leads to CXCR4 upregulation. The next step is to enter the bone marrow and to undergo apoptosis (66). In carbon tetrachloride (CCl_4_)-based liver injury, cell therapy with bone marrow-derived macrophages (BMM) resulted in hepatic recruitment of endogenous macrophages and neutrophils. These cells were found to express matrix metalloproteinases-13 and -9, respectively, as noted in scar regions. The cell infiltrate also expressed increased levels of the antiinflammatory IL10 (67). Elevating the neutrophil count using i.e., the drug Filgastim [Non-glycosylated granulocyte-colony stimulating factor (G-CSF)] also improves the survival of patients with severe alcoholic hepatitis: and acute liver failure (68).

The release of ROS by neutrophils has been reported to mediate macrophage phenotype in mice switching from pro-inflammatory Ly6C^hi^CX_3_CR1^lo^ to pro-resolving Ly6C^lo^CX_3_CR1^hi^ macrophages, thus contributing to tissue repair in APAP liver injury model (69). NETs are released under high neutrophil densities aggregate and degrade cytokines and chemokines via serine proteases, thus promoting the resolution of neutrophilic inflammation in a mouse gout model (70). The beneficial effects of neutrophils in clearance of cellular debris during sterile inflammation were outlined clearly before (66). The effects of neutrophil fate on macrophage activation is summarized below in section Impact of Neutrophil Fate and Activities on Macrophage Polarization and deserves special attention, due to the important role of macrophages.

These studies suggest that neutrophils can play opposing roles for the outcome of inflammation. While they can amplify the inflammatory cascade i.e., by ROS or NETs, they are also able to support the resolution of inflammation, i.e., by MMP9. The contribution of neutrophils depends on the type or model and the specific interactions with other hepatic cell types (Figure 1B) (71).

### Neutrophils in Chronic Liver Disease

Fibrosis can develop in the liver as a consequence of continuous liver injury and occurs in most types of chronic liver diseases. A prominent feature in chronic liver diseases is the accumulation of neutrophils. Compared to acute liver diseases, contributions of neutrophils in chronic liver injury are less clear. An *in vitro* study suggested that neutrophil-derived ROS stimulates collagen synthesis by human hepatic stellate cells whereas neutrophil-derived nitric oxide may exert a protective antioxidant effect by operating as a scavenger of superoxide anion (72). It is therefore intriguing to investigate how neutrophils orchestrate the release of these two products *in vivo* during liver fibrosis. Neutrophils from patients with chronic liver disease showed abnormal adherence to nylon fibers *in vitro* irrespective of the underlying etiology (73), whereas other functions such as phagocytosis and killing were rather normal (74). However, a more recent report revealed that stable cirrhosis is characterized by a malfunction of neutrophil phagocytosis, and their secretion of increased amounts of inflammatory mediators (75). The anti-neutrophil cytoplasmic antibodies (ANCA) are another hallmark of advanced fibrosis; it is a class of antibodies which binds to several different targets in neutrophils. Enhanced ANCA immunoglobulin is a feature of cirrhosis regardless of its etiology, and is significantly increased in patients with cirrhosis (in alcoholics and non-alcoholics), but not in healthy controls or HCV patients. Thus, levels of ANCA immunoglobulin A (IgA) increase with disease progression (76).

There is a huge proportion of people with fatty liver disease in the industrialized countries. Liang et al. (77) have systematically compared the role of inflammatory and metabolic triggers on the development of non-alcoholic steatohepatitis (NASH), a disease which often precedes liver fibrosis. Mice treated with metabolic dietary triggers (carbohydrate, cholesterol) developed NASH, characterized by enhanced steatosis, hepatocellular hypertrophy, and formation of mixed-type inflammatory foci containing also myeloperoxidase-positive granulocytes (neutrophils), in addition to mononuclear cells, essentially as observed in human NASH. In contrast, non-metabolic triggers such as lipopolysaccharides (LPS) and interleukin-1β (IL-1β) did not induce a NASH-like phenotype. MPO aggravates the development of NASH and increase adipose tissue inflammation in response to a high fat diet and thereby plays an important role for neutrophils in the pathogenesis of metabolic disease. (78). Interestingly, the ROS production by monocytes was similar in NASH patients and healthy controls, while the neutrophils exhibited a particularly higher phorbol myristate acetate-induced production of ROS (79). Data from a mouse model of high fat diet (HFD) with binge ethanol feeding have shown that obesity and binge drinking are synergistic in promoting liver fibrosis, and this process is partially mediated through interactions between neutrophils and hepatic stellate cells (HSC). The authors have shown that neutrophils activate HSC. Vice versa, HSCs produce granulocyte-macrophage colony-stimulating factor and interleukin 15 which support the survival of neutrophils (Figure 1B) (80).

### Neutrophils in Hepatocellular Carcinoma

Hepatocellular carcinoma (HCC) is one of the most common malignant tumor types in the world. Recently, it was shown that in non-alcoholic steatohepatitis, elevated free fatty acids stimulate NET formation and promote the development of HCC, suggesting a role of neutrophils in the progression of liver cancer in NASH (81). The number of neutrophils in peripheral blood strongly associates with prognostic outcome, indicating a potentially distinctive role for neutrophils as facilitators of tumor progression and deteriorating performance (82). Mechanistically, it was shown that neutrophils may promote angiogenesis in the tumor milieu as a major source of MMP-9. (83). Another study showed that tumor associated neutrophils (TAN) mediated the intratumoral infiltration of macrophages and T_reg_ cells by secreting CCL2 and CCL17, which stimulated neovascularization, enhanced HCC growth and metastasis in a mouse model, as well as in HCC patients (84). In particular, the ability of neutrophils to suppress T cell proliferation has increased recognition of neutrophils and brought a new name for these cells as granulocytic myeloid-derived suppressor cells (gMDSC). Updated nomenclature has re-defined these cells as myeloid regulatory cells (MRC) (85).

The granulocytic MRC have received increasing attention from scientists. Many patients with advanced cancer suffer from neutrophilia, and TAN are associated with poor prognosis. Recently, the neutrophil-to-lymphocyte ratio has been proposed as a novel prognostic factor in many types of cancer. However, the actual role of neutrophils for tumor development is controversial, and there are example for pro as well as anti-tumoral functions, as reviewed by Shaul and Fridlender (86) the transforming growth factor β (TGFβ) triggers neutrophils to be pro-tumoral. In turn, TGFβ blockade results in the recruitment and activation of TANs with an antitumor phenotype, described as “N1” vs. “N2” TAN by Zvi Fridlender (87). The N1 and N2 neutrophils both are capable of producing NETs, but their individual roles remain enigmatic. Christoffersson et al. have demonstrated that inflammatory neutrophils (CD11b^+^ Gr1^+^CXCR4^low^) get recruited to sites of injury by CXCL2 whereas angiogenesis promoting (MMP9^hi^ CXCR4^hi^) are recruited by the presence of vascular endothelial growth factor A (Vegfa). Their important role in vascularization strongly suggests that these pro-angiogenic neutrophils exhibit the N2 subtype (88).

Recently, a close interrelation of neutrophils and macrophages has been demonstrated in different cancer models. In particular, depletion of macrophages using antibodies which target colony stimulating factor 1 (CSF1) leads to a compensatory activation of neutrophils, which is induced by cancer-associated fibroblasts. Thus, an additional inhibition of neutrophils, in parallel to that of macrophages has demonstrated increased treatment efficiency (89). Furthermore, the specific properties of dendritic cells have to be taken into account for advanced treatment targeting neutrophils (90).

### Impact of Neutrophil Fate and Activities on Macrophage Polarization

Upon their arrival in tissues, neutrophils become apoptotic and get removed by macrophages and dendritic cells though phagocytosis. Senescent neutrophils in blood are known to upregulate CXCR4 which instructs them to return into the bone marrow for clearance (91). This clearance further is of importance for controlling neutrophil generation in the bone marrow (92). The uptake of apoptotic neutrophils by macrophages triggers an anti-inflammatory response and polarization of the macrophages, i.e., a reduction in IL23. The reduction in IL23 leads to reduced IL17 levels and thus, also reduced G-CSF. The final consequence of this cascade leads to diminished granulopoiesis (92).

However, if neutrophils receive a survival signal from inflammatory mediators, they can activate macrophages which can amplify inflammation. In contrast to neutrophils, the maturation of macrophages from monocytes begins only after they have entered the tissues. MΦ play crucial roles in controlling inflammation in different organs. The tumor necrosis factor (TNF) is a key factor generated by MΦ and covers a broad line of biological functions, and in many cases, is involved into cell and organ injury. During inflammatory diseases, TNF can lead to necroptotic parenchymal cell death. Many types of parenchymal cells of different organs are sensitive to MΦ-derived TNF, thus fueling the disease, for instance, in liver inflammation (42), rheumatoid arthritis (93), autoimmune disease (94), and psoriasis (95).

**Figure 2:**
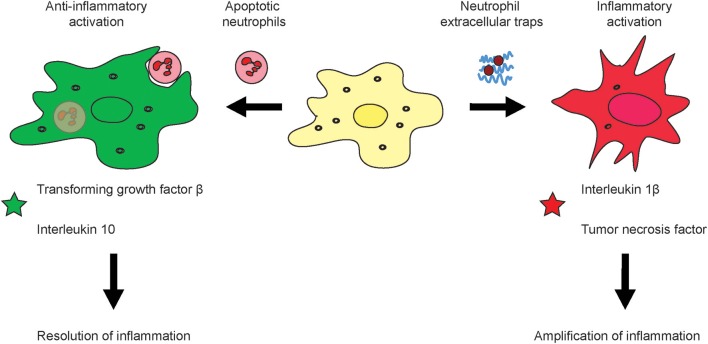
Effects of neutrophil activation and viability on macrophage activation. During the course of inflammation, neutrophils can response in different fashion to their activation i.e., by pathogens or sterile stimuli such as DAMPs. It is known that apoptotic neutrophils induce alternative activation of macrophages which leads to an anti-inflammatory subtype which produces substances which down-regulation inflammation and support healing of tissue such as the Transforming growth factor β and Interleukin 10 **(left)**. Upon excessive inflammation, NETs can amplify tissue injury by means of their secreted molecular factors such as the tumor necrosis factor or Interleukin 1 β **(right)**.

Targeted depletion of MΦ can be done by using particulate carriers such as micron-sized clodronate liposomes which MΦ scavenge actively. The clodronate of these micro-sized lipid carriers is, upon uptake, released intracellularly and induces apoptosis of MΦ. However, blocking of tumor-associated macrophages (TAM) infiltration into tumors by inhibiting CSF1R signaling has a limited level of success, which is very likely explained by a mechanism that CSF1R inhibition unleashed the expression of granulocyte-specific chemokine in carcinoma associated fibroblasts, thus resulting in infiltration with tumor-associated neutrophils (TAN) (89), also referred to as polymorphonuclear myeloid-derived suppressor cells (PMN-MDSC). Upon inhibition of CSF1R in cancer, an increased amount of TAN is recruited into the tumors which compensate a lack of TAM or monocytic MDSC. The solution to overcome this myeloid cancer immune escape might be to combine inhibitors for TAM (CSF1R-inhibitors) and TAN (CXCR2-inhibitors) (89). Targeting of toll-like receptor (TLR) signaling is approved for melanoma treatment. Importantly, TLR are differentially expressed by subsets of TAM as recently discovered in own investigations in liver cancer, and the cellular effects on myeloid cells remain to be understood in more detail (96, 97). In summary, the fate of neutrophils has a major impact on macrophage activation and may lead to a polarization into either rather inflammatory or anti-inflammatory macrophages (Figure 2).

### Identification of Novel Mechanisms Controlling Migration and Activation of Neutrophils

The last decade has brought increasing attention by scientists for neutrophil granulocytes. Novel tools have evolved from knockout mice strains which allow to study different neutrophil subsets in parallel. For many years, it was difficult to label neutrophils specifically, while they are present in interstitial tissue. The common fluorescent tags and the frequently employed LysM reporter strain have problems in differentiating neutrophils from monocytes/macrophages. In particular, difficulties come up in inflamed tissue sites, which are characterized by a strong presence of all phagocytes. A novel mouse strain which uses the more specific promoter Ly6G, which is exclusively expressed in neutrophils, has been developed (98). The novel strategy thus enables to specifically and unequivocally study neutrophils in organs of living organisms (66).

Importantly, in the mouse, neutrophils only account for 10–25% of peripheral blood cells. Additional differences consider several important aspects such as the genomic response (99). Primary neutrophils are prone to pre-activation, but there is no ideal alternative to studying primary cells since neutrophil-like cell lines do not reflect the functionality and diversity of these cells (100). Thus, systematic studies on neutrophil therapies should include both human cells in *in vitro* settings and mouse or other rodent in *in vivo* models to gain a maximum level of translation. However, due to the rapid activation of neutrophils *in vitro*, such data have to be carefully interpreted.

Two photon microscopy (2PM) enables to quantify and readily visualize live cell behavior *in vivo*. This cutting-edge technology allows to study cell behavior in specific tissues, interaction with one another, as well as how cells respond individually and collectively to therapeutic intervention (101). Liver is not only the most important metabolic organ but also a central axis in the immune system. In the last decade, many intravital imaging studies in both basal and pathological conditions have revealed the complexity and tissue specificity of immune responses within the liver (102). It was observed that in ischemia and reperfusion (I/R) injury, neutrophil infiltration and platelet adhesion in presinusoidal arterioles and post-sinusoidal venules contributes to liver damage. Intravital imaging has also been used to visualize and quantify liver necrosis in APAP induced acute liver injury, the same study revealed that neutrophils accumulate in great numbers in the liver and subsequently migrate specifically to the interior of necrotic zones at the peak of APAP toxicity. In Concanavalin-A (Con-A) induced liver injury, neutrophils are directly activated by Con-A and neutrophil recruitment precedes T cell arrival. Advanced intravital imaging has been also applied to facilitates dynamic monitoring of cellular and molecular activity upon therapeutic intervention. Such imaging approaches have also been used to study *in vivo* pharmacology at the single-cell level (103). Traditionally, drug pharmacokinetics (PK) have been analyzed by methods such as mass spectrometry, which only reflect PK properties in bulk tissues and fluids, whereas intravital imaging allows dissection into cellular and molecular levels, as well as heterogeneity across individual cells. So far, the majorities of such studies have been focusing on cancer and provide important information on how drugs work and fail *in vivo*.

### Routes for Targeting Neutrophil Recruitment, Migration, and Activation

#### Therapeutic Induction of Bone Marrow Egress of Neutrophils

**Figure 3:**
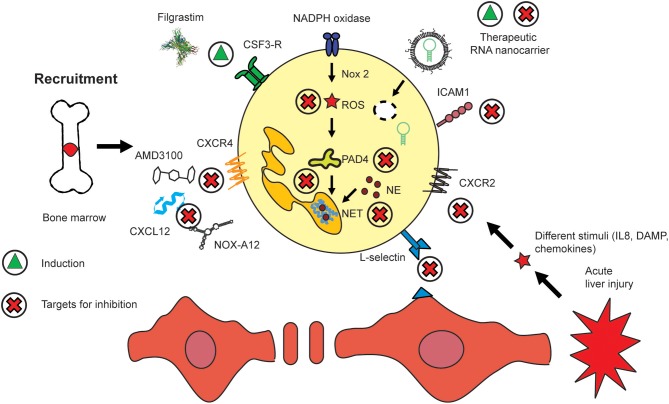
Routes to modulate hepatic neutrophil recruitment, activation, and migration. The presence of neutrophils has been shown to be beneficial i.e., in sepsis where they are mobilized by recombinant CSF3 (Filgrastim). Similar effects are exerted by CXCR4/CXCL12 inhibitors such as AMD3100 and NOX-A12. Nanomedicines have been employed to deliver artificial micro-RNA inhibitor targeting miR223. Key molecules which regulate the activation of neutrophils include those pathways which lead to NET and ROS generation. The migration of neutrophils into inflamed tissue sites can be inhibited by targeting their adherence to the endothelium, i.e., by selectins such as L-selectin or integrins, i.e., ICAM1. The induction of a compound or mediator is indicated by a green triangle, and inhibition by a red cross.

Neutrophils are motile leukocytes which are mobilized from the bone marrow and migrate into inflamed tissue sites based on different stimuli, i.e., chemokine gradients. On their way to their target, their functionality can be modulated with appropriate drugs acting on key mediators for these different fields. Thus, the therapeutic strategies which exist today mainly to focus on either neutrophil recruitment, migration or activation. In fact, all processes are functionally linked closely and certain drugs might act on different areas in parallel. The continuous influx of neutrophils at huge numbers during acute inflammatory settings exhibits a huge, yet mostly unexplored potential for interventions. Nowadays, the most important therapy which aims at neutrophils makes use of the immunostimulatory properties of the recombinant granulocyte colony stimulating factor (G-CSF, CSF-3) Filgastrim (104). Increasing the numbers of neutrophils was shown to be beneficial for different kinds of disease, for instance, these cells have impaired bactericidal functions (sub-)acute liver failure, similar to that seen in severe sepsis (105). Therapeutic administration of filgrastim can assist in reversing defective neutrophil functions and can prevent liver failure. The small molecule AMD3100 (Plerixafor, molecular weight of 503 Da) is an antagonist of CXCR4 which prevents the accumulation of lymphoid leukemic cells in the protective bone marrow and mobilizes them into blood for improved accessibility by chemotherapy. Thereby, AMD3100 promotes the efficacy of chemotherapeutic agents (106). A similar and potentially alternative strategy for neutrophil (and lymphocyte/monocyte) translocation from bone marrow into blood might be given by NOX-A12. It is an RNA oligonucleotide which binds and neutralizes CXCL12. It thereby removes chronic lymphoid leukemia cells from the protective microenvironment of the bone marrow. It also acts on mature neutrophils which express CXCR4 (Figures 1A, 3). The compound has been studied in chronic lymphocytic leukemia (CLL) where it was reported to interfere with cell chemotaxis and stroma-mediated drug-resistance. The corresponding treatment is also immunostimulatory since it mobilizes white blood cells, in particular lymphocytes, neutrophils, and monocytes, into circulation (107).

#### Targeting Endothelial Transmigration of Neutrophils

The transmigration of neutrophils to sites of inflammation is guided by adhesion molecules which function as molecular traffic signs (108). These molecules are represented most prominently by selectins and integrins. Despite the minor role of selectins and other adhesion molecules for liver recruitment of neutrophils (44), targeting selectins has been proposed for a wide variety of inflammatory disorders of other organs. These diseases include post-ischemic injury, brain, lung, heart, and skin inflammation, atherosclerosis, and cancer (109). For instance, selectin-blocking agents are already in clinical trials to treat chronic obstructive pulmonary disease (COPD) (110) and sickle cell disease (111). Using miniaturized selectin-directed nanomedicines (NM), we demonstrated binding of different variants of sulphated selectin-binding groups based on structural similarity with selectins (112, 113). The process of neutrophil attachment and transmigration through the endothelium requires an interaction of CD11b and/or CD18 integrin expressed by neutrophils, with ICAM-1 which is located at the endothelium (114) correspondingly, the inhibition of the C-X-C chemokine ligand 1 (CXCL1) or of the intercellular adhesion molecule-1 (ICAM1) reduced hepatic neutrophil infiltration and ameliorated liver injury and fibrosis (80). Blocking of neutrophil infiltration by anti-granulocyte receptor 1 (Gr-1) depletion or combined CXCR2–formyl peptide receptor 1 antagonism was demonstrated to significantly reduce hepatocyte injury in mice (115). Despite the minor role of selectins for neutrophilic liver recruitment, we have revealed that blocking selectins with specific nano-sized inhibitors significantly affects the outcome of liver injury (112).

The activation of neutrophils in inflammatory diseases should be reduced by therapeutics and potential treatment options may result from an induction of wound healing properties of neutrophils. It might also be useful to simply trigger neutrophil apoptosis to induce wound healing activities of macrophages (71). Wound healing is often associated with alternative activation of macrophages, as recently demonstrated in a human skin model in own studies (116). The inhibition of TNF might additionally or alternatively help to induce a wound healing phenotype of neutrophils and macrophages (93). Recently marketed small molecule-based drugs still block the mechanism of action of certain inflammatory cytokines. In particular, tyrosine kinase inhibitors have made it into the collection of anti-inflammatories. The Janus kinase (JAK) inhibitors, for instance, intend to block cytokine signaling including that of TNF. For instance, Tofacitinib, a small molecule-based pan-JAK inhibitor (312 Da) received approval for diverse inflammation-related diseases such as inflammatory bowel disease (117), psoriasis (118), and arthritis (119). However, neither TNF inhibitors such as infliximab nor JAK inhibitors improve the outcome of liver diseases.

#### Nanomedicines for Modulating Neutrophil Functions

Small molecules have the major advantage of high body and tissue penetration. However, their drawback is their systemic distribution which can affect off-target sites. Generally, Nanomedicines (NM) are nano-sized drug carriers which can be used clinically. NM offer many possibilities to improve drug delivery. Organic nanomaterials are most appropriate for clinical applications since most of them are biodegradable. The most popular base material for organic NM are liposomes. Liposomes are empty artificial vesicles composed mainly of phospholipids that also are a main constituent of natural cell membranes (120). Upon loading with a drug, loaded liposomes should be referred to as lipid-based nanocarrier. Inorganic nanomaterials such as gold nanoparticles (AuNP) have also been evaluated for clinical applications, but particularly AuNP have the drawback that they accumulate in the body, especially in the liver (121). Iron oxide nanoparticles (ferum oxytol) are clinically approved for treatment of anemia and are popular as contract agent (122). We have conjugated AuNP to small molecule-based drug stavudine and thereby have increased the uptake of stavudine by macrophages (123). In practical terms, a biologically active molecule, i.e., a small molecule can be encapsulated, for example, by a lipid membrane, or conjugated with albumin to render drug pharmacokinetics in a desired fashion. Referring to the market volume, Doxil is the most successful nanomedicine. It was approved in 1995 and contains doxorubicin, a chemotherapeutic drug, which is encapsulated in a lipid carrier (124). It protects the body from systemic exposure to the toxic chemotherapeutic drug which can also harm healthy tissue in the body. Thus, NM can protect the body from the drug and the drug from the body. NM also enable to combine several features in a single carrier. For instance, the NM surface can be equipped with a ligand for cell receptors which serves as a targeting vector whereas the core can be loaded with a drug, allowing for selective delivery of tailored drugs (108). This specific drug targeting, i.e., by using a ligand specific for a certain receptor, is referred to as “active targeting.” Albumin and lipid-based NM belong, in terms of market value, to the most successful types of NM formulation.

Importantly, the most relevant mechanism for tumor targeting is “passive targeting” based on the enhanced permeability and retention (EPR) effect (124). Active targeting of neutrophils might be achieved by aiming at neutrophil-specific receptors such as CD15 or by engaging L-selectin (112). The outstanding mobility of leukocytes can also be used for another strategy in which immune cells are used to transport nanoparticles to the desired site of action—as Trojan horses. A promising study shown by Choi et al. illustrated that monocytes might be used to destroy hypoxic tumor regions by their delivery of cytotoxic compounds (125). Similar strategies might also make use of the high numbers of neutrophils to deliver drugs into tumors or inflammatory sites. It might for instance be thinkable to trigger neutrophils to release a drug which is coupled to a neutrophil receptor. For instance, Doxil, a chemotherapeutic drug might be coupled to the general neutrophil receptor CD15, or to one of the other neutrophil receptors, i.e., CXCR2, CXCR4, ICAM1, or L-selectin. Virtually, the delivery through all receptors shown in Figure 1A is possible in theory. The release of a drug might be enabled by a cleavable linker, which can for instance be loosened from the cells by pH-dependent cleavage. The drug would accordingly be transported into the tumor. In an analog fashion, neutrophils might also be used to transport anti-inflammatory drugs toward inflammatory lesion. However, this is a speculative concept which has not been studied experimentally.

The therapeutically most important molecular pathways of neutrophils are most likely those that lead to the formation of ROS and NETs, both of which are linked through a positive feedback loop via Pad4, a nuclear enzyme which mediates NET formation by histone hypercitrullination and thereby contributes to chromatin decondensation (Figure 3) (126). There have been few attempts to use nanomedicines for modulating neutrophils. One example, has been targeting ROS generation by aiming at nicotinamide adenine dinucleotide phosphate hydrogen (NADPH) oxidase in macrophages using an siRNA against Nox2, a catalytic subunit of NADPH oxidase. However, the cellular effects have only been studied in macrophages (127). However, there were also reports that targeting of NADPH oxidase exacerbates rheumatoid arthritis in mouse models (128). The NE which is found in NETs has also been targeted, and was found to solubilize sputum. NE is inhibited in the course of palliative treatment of cystic fibrosis patients (129). Neutrophils have been targeted using Piceatannol-loaded Albumin-based NM to detach them from vasculature, which prevented vascular inflammation (130). Our own recent studies have analyzed the potential of modulating neutrophil migration through interference with selectin-binding ligands, a concept that even avoids the attachment of neutrophils to the endothelium during the earliest stage of transmigration. We have recently studied the effects of selectin-directed glycopolymers on the migration and activation of neutrophils and other immune cells on liver injury. *In vitro* migration and *in vivo* migration were selectively affected by specific selectin-binding groups and were linked the outcome of inflammatory liver injury (112, 113). Using nanoparticles to target immune cells might also target LSEC since they express scavenging receptors such as the mannose receptor (131). Interestingly, the scavenging function of LSEC appears to not account for nanoparticles as we did not locate gold nanoparticles in LSEC using transmission electron microscopy, but exclusively in macrophages (121).

Liposomal dexamethasone (Lipodex) serves as an interesting example for improving the pharmacokinetics of a drug by nanotechnology: while free dexamethasone distributes systemically to virtually all accessible parts of the body, encapsulation protects many cells from its cytotoxicity. Lipodex further increases targeting of the corticosteroid to liver cells, thus Lipodex mostly translocates to the liver. The encapsulation has a strong impact on toxicity, for example, human fibroblasts and MΦ are protected from the cytotoxic effects of free dexamethasone *in vitro* studies (120). *In vivo*, Lipodex efficiently targets immune cells resulting in therapeutic effects: at a concentration of 1 mg/kg body weight, autoimmune-mediated liver disease triggered by Concanavalin A was significantly reduced by Lipodex, but not by free dexamethasone at the same dose (132). The benefits of drug encapsulation are similarly improved for the chemotherapeutic drug doxorubicin which half-life is extremely short (few minutes) and which can be extended by liposomal formulation for up to several days (133).

Assumingly, all drugs and NM have certain immunomodulatory effects. There is a huge number of NM available which being of either organic, inorganic, or hybrid nature. Many of the different properties, such as material type, size, and functionalization have an impact on the distribution in the body and accumulation in different cells. The distribution to different organs is known to strongly depend on the size of the particles. For instance, particles sizing from 10 to 250 nm mostly distribute to liver and spleen. Smaller sizes allow for a localization to also other organs than liver. Specifically, those below 10 nm increasingly translocate to kidney, testis, and brain (134). The most intensively studied inorganic NM probably are gold nanoparticles (AuNP), which can be easily modified in size, shape, and functional endgroups. This ease of use allows for example for peptide-decoration at the surface (135). Earlier studies have demonstrated that nanoparticle surface chemistry can affect the amount of particles trapped in NET (136). It has to be taken into consideration that inorganic nanoparticles accumulate for certain periods in the body since they are not biodegradable. This has been shown for the first time in own studies by non-invasive imaging based on computerized tomography. In this study, gold nanorods retained at similar levels in the liver 1 week after injection (121). There were few clinical trials facilitating gold nanoparticles, one was on CYT-6091, a gold nanocarrier for TNF with level I clinical completed in cancer therapy (137).

Modulation of neutrophils with nanomedicines should take opportunities and risks into account. In addition to using surface immobilized ligands for active targeting, such as those targeting selectins (112). NM in parallel allow for a modulation of neutrophil activation by delivering appropriate drugs targeting corresponding neutrophil pathways. Importantly, while lymphocytes, monocytes, and macrophages respond well to liposomal and free Dexamethasone with reduced cell numbers *in vivo*, neutrophils were unaffected by the corticosteroids (102, 113). Therefore, there is a high need to modulate neutrophils in an improved fashion by aiming at their specific pathways such as ROS or NETs (Figure 3). It also has to be taken into account that a modulation of chemokines and their receptors generally exhibits a limited level of cell type specificity because also many other cell types express chemokines and their receptors, i.e., also hepatic stellate cells express CXCR4. Nevertheless, chemokine modulation mostly affects the motile immune cells. One advantage of chemokine-directed therapy is the option to target them by using small molecules, such as Cenicriviroc, which blocks the CC-chemokine receptors CCR2/CCR5 and which is now in phase three clinical trials on liver fibrosis (138).

A more selective and specific modulation of immune cells by a cell type specific cargo, i.e., by nanomedicines delivered via phagocytic uptake might increase efficiency of therapeutics. NM offer the opportunity to be delivered spatially; i.e., magnetic nanocarriers can be concentrated in specific areas of the body using magnetic fields (139). Thermodox, which enables a heat-sensitive release of doxorubincin, is another example for a localized drug delivery for patients with liver cancer (140). Heat-induced drug release might also be adapted to the field of anti-inflammatory therapies to enable a localized release of anti-inflammatory drugs in selected organs.

Furthermore, it is important to be aware of the risks of neutrophil modulation, particularly in multimorbidity. For instance, it might be useful to inhibit the inflammation amplifying functions of neutrophils in inflammatory disease, but this at the same time might promote tumor growth (87). The margination of the neutrophil pool further has to be considered for advanced therapies (10). Neutrophils might also be mobilized from the marginated sites, what thus bears an additional therapeutic potential. However, a disturbed balance of the neutrophil pool might lead to problems in some of the organs where the marginated cells are found, such as liver and bone marrow, particularly, under inflammatory conditions. A lack of these cells might lead to problems in combating infections, whereas increased numbers might also lead to unspecific tissue injury.

Nevertheless, it has to be considered that inflammatory conditions might affect the phagocytic capabilities of neutrophils: aged neutrophils were discovered to exhibit an increased phagocytic uptake compared to non-aged neutrophils (141). Therefore, the capabilities of targeting the different subsets of neutrophils with nanomedicines has to be analyzed in great detail, meaning that for flow cytometry, nanocarriers should be labeled with fluorescent tags and be combined with fluorescently labeled antibodies, as reported before in own studies (132). It might also be conclusive to study the interactions of platelets and monocytes with neutrophils (57).

#### Microfluidic Production of RNA-Based Drugs

Small non-coding RNA (sncRNA) such as small interfering RNA (siRNA) and micro-RNA (miR) offer an enormous spectrum of molecular tools for advanced reprogramming of neutrophils. SncRNA exhibit enzymatic functions: while siRNA is able to inhibit a single mRNA, miR modulators such as the single-strand inhibitory antagomirs can up and down-regulate multiple targets in gene transcription and translation (142). The so-called pro-miR can induce the abundance of an endogenous miR by stimulating its production. Notably, miR is known to regulate at least 60% of all protein-coding genes (143). Certain miR(s) regulate complex gene regulatory networks by acting on different target sites in parallel. Despite of their short lifespan, modulating neutrophil activation by a sncRNA was demonstrated to be efficient (144). The miR223 was demonstrated to be an important regulator of neutrophil activity and the skin model was used to study therapeutic usability of an antagomir for miR223. Upon its inhibition, skin wounds healed faster, and chronic inflammatory processes were prevented. Interestingly, neutrophils from miR223 deficient mice further showed increased expression of MPO and ROS (144). MiR was shown to play an important role in the regulation of inflammatory liver disease, as demonstrated by Roy et al. (145). Thus, the modulation of sncRNA such as miR expression is a useful tool for novel and efficient NM and can be used to either inhibit or induce the expression and/or translation of target mRNA/proteins. The conceptional design of sncRNA using drugs should settle on top of the knowledge databases of the “omics” sciences, i.e., genomics, transcriptomics, and proteomics, hereby just mentioning a few key disciplines of molecular biology.

The stability of free sncRNA is a major pitfall in designing corresponding drugs. The stability of sncRNA *in vivo* is limited and therefore, therapeutic applications of sncRNA require sequence modifications for achieving efficient delivery of siRNA or miR. In detail, nucleotide analogs with constrained furanose ring conformations such as locked nucleic acids (LNA) lead to increased binding to the RNA target and improve stability toward nucleases. The LNA monomer contains an O2'–C4' linkage which keeps the furanose ring in N-type. In contrast to LNA, unlocked nucleic acid (UNA) is an acyclic analog of RNA with the only difference of cleavage between the C2' and C3' bonds (146). The UNA decreases binding to a complementary DNA strand (119). Oligonucleotides which contain cyclic inter-residues such as UNA are reported to amplify RNase H-derived cleavage of complementary RNA if it is applied with so-called gapmers (147). A third example is 4′-C-hydroxymethyl-DNA which overcomes degradation by RNase H (147). In addition to sequence polishing, target cell specificity might be increased by liposomal (nano)carriers or alternative vehicles. This protects the sequence from degradation and reduced effects on non-target cells. Neutrophils belong to the phagocytic cells and the phagocytic activity therefore can be used to deliver sncRNA to them (Figure 3).

In addition to the requirements in sncRNA stabilization methodology, the delivery remains a key issue in RNA medicines. The use of positively charged lipids (or polymers) is the most widely used concept for RNA delivery. However, these so-called lipoplexes or polyplexes are known to exhibit certain degree of cytotoxicity by their positive charge (148). The problem of lipoplexes is the fact that RNA and positive charges can be recognized by immune cells, i.e., by their TLR. Thus, encapsulating RNA might help to reduce cytotoxicity by making the content unrecognizable to the cells. Importantly, these nanocarriers exhibit a neutral surface charge. Lipid-based nanocarriers generated using microfluidics technology were reported to exhibit efficient *in vivo* delivery of RNA to hepatocytes (liver parenchymal cells) (149). The first siRNA-based drug which is used in the clinics, Patisiran, is generated by microfluidic mixing (150). Microfluidic-based production of RNA medicines many advantages compared to conventional methods for generating lipid-based nanocarriers, i.e., the film method, which requires heating (120), which can impact the integrity of drugs to be encapsulated. Microfluidic mixing is based on mixing lipids in a solvent such as ethanol, and RNA in a buffer system with acidic pH such as acetate buffer with pH 4. The size of the nanocarriers can simply be tuned by altering the flow rate of mixing, or the lipid mixtures used (149). In conventional method, extrusion filtration is required to reduce the size of liposomes (120). Studies have outlined that CXCL12 also regulates the differentiation of monocytes and macrophages in liver cancer (151). Liu et al. have used CXCR4-directed nanocarriers loaded with siRNA against the vascular endothelial growth factor (Vegf) as an alternative antiangiogenic therapy and received a potent antitumor response by their carriers, by acting on monocytes, but probably also on neutrophils (Figure 3) (152).

#### Off-Target Effects of Neutrophil-Directed Nanomedicines

Notably, it is difficult to overcome the uptake of neutrophil-directed nanocarriers which i.e., target ROS or NET also by other immune cells such as macrophages or dendritic cells. The most important caveat in this regard is to select a carrier, and also a drug, which has no or only minor effects on the other phagocytic cell types. In the ideal case, also owed to the similarity to the other myeloid-derived phagocytes, the neutrophil-directed drugs may also dampen inflammation by targeting similar function in these other myeloid cells, which to a minor extent, such as monocytes, can also release NETs (136), or express Cathepsin G and elastase 2, as shown for macrophages under specific conditions. Inhibition of Nox2, which is a reliable target in neutrophils, also in macrophages led to beneficial effects in heart failure treatment in the mouse model (127). Particularly, a drug aiming at neutrophils should not lead to tremendous macrophage activation and it should also not influence antigen processing by dendritic cells. We have for instance shown before that peptide-modified gold nanoparticles impact the expression of costimulatory molecules by human dendritic cells (120). In the context of treating inflammatory disease amplified by neutrophils, it should be considered that the modulation of inflammation critical factors such as the TGF-β may also impair the antitumor functions of these cells (87). Therefore, the broad spectrum of cytokines, but also lipid-based mediators which might be modulated by novel neutrophil-directed drugs should be analyzed to identify potential sources for side effects.

Immune cell-directed nanomedicines might also act on other cell types such as hepatocytes, hepatic stellate cells, as well as on endothelial cells (153). It is known from implantology that unintended interactions of materials with innate immune cells can trigger chronic inflammatory disease, lead to tissue damage, and implant rejection (154). Similarly, other materials that are considered as body-foreign and as a threat to the body might be attacked by immune cells and hence induce adverse reactions. There have been different attempts to reduce unspecific interactions with phagocytes and endothelial cells, the most frequently performed strategy probably is to reduce unspecific binding to proteins and cells by functionalization with polyethylene glycol (PEG), referred to as PEGylation (155). However, many researchers are not taking into account that PEG periodically accumulates in parts in the body and that the immune system produces antibodies against PEG. These anti-PEG antibodies are responsible for the so-called accelerated blood clearance (ABC) effect where liposomes are cleared by phagocytes upon repeated administration (156). There are also potentially upcoming alternative polymers which might replace PEG in future, such as poly-sarcosine which shows a similar behavior like PEG. Polymer chemists have demonstrated that the binding potential of poly-sarcosine to albumin, the most frequent human serum protein, is very similar to that of PEG. In addition, the adsorption kinetics and polymer conformation changes showed only minimal differences between PEG and poly-sarcosine (157).

Alternative strategies to inhibit undesired reactions with the RES are optimizing the immune response using coatings that reduce the acute immune reaction. Researchers have demonstrated that s poly(N-isopropylacrylamide) hydrogel particles crosslinked with poly(ethylene glycol) diacrylateridges have such effects (158). Others have suggested using ultrasmall nanogels sizing only two nanometers, which is below the size of phagocytic clearance of these particles (159). Studies of our group have underlined the important of myeloid phagocytes compared to endothelial cells, which internalize large amounts of nanoparticles *in vitro* when cultured as a monolayer (160). Notably, *in vivo*, endothelial cells were not found to contribute to the uptake of gold nanoparticles inside the liver at all, but nanoparticles were found in hepatic MΦ only (121).

## Conclusions

Neutrophils have for many years been underappreciated as bystander cells which mostly function through killing. However, the last decade has shed light on their plasticity and role in different diseases, such as inflammatory disorders and cancer. Currently, neutrophils are not yet in the focus of advanced immunotherapies, and additional novel basic research may help to unravel new concepts for neutrophil-based treatment. Few nanomedicines have been tailored for neutrophils, but existing studies have demonstrated feasibility of miR modulation in these cells. Using novel tools for sncRNA delivery and exploitation of the “omics” sciences such as genomics, transcriptomics, proteomics, and metabolomics using novel biomarkers may advance the field. Nanomedicines may further be multifunctional and theranostics allow for combined therapeutic and diagnostic purpose. Nanomedicines have already improved drug delivery, reduced side effects of chemotherapeutics. Nevertheless, tailored targeting groups and smart features have to be reproducible, simple and cost-efficient to have the chance of being successful in the clinics (161).

## Author Contributions

MB and JW wrote the article.

### Conflict of Interest

The authors declare that the research was conducted in the absence of any commercial or financial relationships that could be construed as a potential conflict of interest.

## Abbreviations

2PM: Two photon microscopy
ABC: Accelerated blood clearance
ANCA: Anti-neutrophil cytoplasmic antibodies
ATP: Adenosine triphosphate
AuNP: Gold nanoparticles
BMM: Bone marrow-derived macrophages
CLL: Chronic lymphocytic leukemia
COPD: Chronic obstructive pulmonary disease
CSF1: Colony stimulating factor 1
CSF1R: Colony stimulating factor 1 receptor
CXCL12: C-X-C motif chemokine ligand 12
CXCR2: CXC chemokine receptor 2
DAMP: Damage-associated molecular patterns
ECM: Extracellular matrix
EPR: Enhanced permeability and retention
gMDSC: Granulocytic myeloid-derived suppressor cells
G-SCF. Granulocyte colony stimulating factor
HA: Hyaluronic acid
HCC: Hepatocellular carcinoma
HMGB1: High-Mobility-Group-Protein B1
HSC: Hepatic stellate cells
ICAM1: Intercellular adhesion molecule 1
IgA: Immunoglobulin A
IL: Interleukin
IL-1β: Interleukin-1β
JAK: Janus kinase
JAM: Junctional adhesion molecules
Lipodex: Liposomal dexamethasone
LPS: Lipopolysaccharides
LSEC: Liver sinusoidal cells
LTB4: Leukotriene B4
Ly6C, Lymphocyte antigen 6 complex: locus C
miR: Micro-RNA
MMP: Matrix metalloproteinase
MPO: Myeloperoxidase
MRC: Myeloid regulatory cells
Myh9: Myosin heavy chain 9
MΦ: Macrophages
NADPH: Nicotinamide adenine dinucleotide phosphate hydrogen
NASH: Non-alcoholic steatohepatitis
NE: Neutrophil elastase
NET: Neutrophil extracellular traps
NM: Nanomedicines
NM: Nanomedicines
Pad4: Peptidylarginine deiminase 4
PEG: Polyethylene glycol
PMN-MDSC: Polymorphonuclear myeloid-derived suppressor cells
PSGL-1: P-selectin glycoprotein ligand 1
ROS: Reactive oxygen species
SDF-1: Stromal cell-derived factor 1
SHAP: Serum-derived hyaluronan-associated protein
siRNA: Silencing RNA
SIRS: Systemic inflammatory response syndrome
SMC: Smooth muscle cells
Snc-RNA: Small non-coding RNA
suPAR: Soluble urokinase plasminogen activator receptor
TAM: Tumor-associated macrophages
TAN: Tumor associated neutrophils
TGFβ: Transforming growth factor β
TLR4: Toll-like receptor 4
TNF: Tumor necrosis factor
uPAR: Urokinase plasminogen activator receptor
Vegf: Vascular endothelial growth factor.
